# Cytokine profiling of maternal peripheral and umbilical cord blood in term and preterm labor

**DOI:** 10.3389/fimmu.2026.1786565

**Published:** 2026-04-07

**Authors:** Tiantian Yu, Huiling Zhang, Yuan Zhang, Yanan Li, Zixuan Huang, Danyang Lu, Jianxia Fan, Weihong Zeng

**Affiliations:** 1 The International Peace Maternity and Child Health Hospital, School of Medicine, Shanghai Jiao Tong University, Shanghai, China; 2 Shanghai Key Laboratory of Embryo Original Diseases, Shanghai, China; 3 Shanghai Municipal Key Clinical Specialty, Shanghai, China

**Keywords:** chemokines, cytokines, growth factors, immune landscape, preterm birth

## Abstract

Immune dysregulation is increasingly implicated in the initiation of labor and the pathogenesis of preterm birth (PTB), yet the relative immune signatures of maternal and fetal compartments remain poorly defined. We performed multiplex profiling of 48 cytokines, chemokines and growth factors in paired maternal peripheral blood (PB) and umbilical cord blood (CB) collected from 78 pregnancies, including term births, spontaneous PTB (S-PTB), and PTB associated with premature rupture of membranes (PROM-PTB). Our data showed that PB and CB displayed clearly distinct immune landscapes, with CB enriched for immune-regulatory mediators and exhibiting more coordinated cytokine correlation networks, indicative of a highly structured fetal immune milieu. Principal component analysis revealed substantial overlap in global cytokine profiles among term and PTB subtypes in both compartments, consistent with largely conserved immune architecture. Accordingly, cytokine levels in maternal PB remained largely comparable across labor types, with no significant differences observed among term, S-PTB, and PROM-PTB pregnancies. In contrast, CB exhibited pronounced PTB-associated immune remodeling relative to term delivery, characterized by increased expression of growth factors M-CSF and SCF. Beyond these shared changes, S-PTB was further marked by upregulation of IFN-γ, IL-2Rα, MIP-1α, SDF-1α, G-CSF and SCGF-β, whereas PROM-PTB showed broader reductions in eotaxin and HGF. Together, these findings indicate that immune alterations associated with PTB are predominantly reflected in the fetal circulation and may involve distinct immunological mechanisms across PTB subtypes.

## 1 Introduction

Preterm birth (PTB), defined as birth before 37 weeks of gestation, continues to represent a major challenge in perinatal medicine, accounting for a substantial proportion of neonatal mortality and long-term morbidity worldwide (1, 2). Beyond its immediate clinical impact, PTB is increasingly recognized as a biologically complex condition with heterogeneous underlying mechanisms rather than a uniform disease entity (3). This heterogeneity is particularly evident among spontaneous PTB, which comprises distinct clinical subtypes, most notably spontaneous preterm labor with intact membranes (S-PTB) and PTB following premature rupture of membranes (PROM-PTB) (2, 4). Although these subtypes converge on the shared outcome of premature delivery, they differ in membrane status, tissue remodeling and inflammatory context, suggesting divergence in upstream biological regulation.

Labor, whether occurring at term or preterm, is not a passive mechanical event but an actively regulated process involving tightly controlled inflammatory signaling. Endocrine cues and mechanical stretch converge with immune activation to initiate cervical ripening, myometrial contraction, and rupture of the chorioamniotic membranes (5, 6). Cytokines, chemokines and growth factors are integral to these processes, coordinating leukocyte recruitment, extracellular matrix turnover, vascular adaptation and tissue repair (7–11). When these immune pathways are activated prematurely or fail to resolve appropriately, they may precipitate pathological labor initiation and compromise fetal–maternal homeostasis.

Despite sustained interest in inflammation-associated mechanisms of PTB, current understanding remains incomplete. Several studies have examined inflammatory mediators in maternal circulation in association with PTB. For example, multiplex chemokine profiling identified elevated HCC-4 (CCL16), I-TAC (CXCL11), MIP-3α (CCL20) and TARC (CCL17) in maternal peripheral blood (PB) from women with PTB compared with term controls (11). Another study measuring more than 20 cytokines and chemokines reported increased CXCL1 and CCL17 in preterm labor (PTL), whereas CXCL1 and IL-6 were elevated in PROM-PTB (8). Additional biomarker studies reported that low maternal CCL2 levels and elevated CXCL10 or CXCL10/CCL2 ratios are associated with increased risk of early PTB (12), while circulating M-CSF levels have also been implicated in maternal immune adaptation during pregnancy (13).

Beyond maternal PB, several studies have also examined inflammatory mediators in fetal circulation. Multiplex analysis of 27 cytokines in cord blood (CB) identified elevated IL-8, MCP-1 and MIP-1α in association with intrauterine inflammation, PTB, and neonatal complications (10), while another study reported that increased TNF-α levels in CB were linked to a higher risk of PTB (14). In contrast, cytokine profiling studies have reported reduced levels of multiple pro-inflammatory mediators—including IL-1β, IL-6, IL-17A, IL-8, eotaxin, MIP-1α and MIP-1β—in preterm CB, accompanied by increased IL-15 and MCP-1 (15). Proteomic analyses have similarly identified elevated IL-6 and MCP-1, but decreased IL-18 and RANTES in CB from preterm infants (16).

However, most previous studies focused on a limited number of circulating cytokines or chemokines and therefore may not fully capture the complexity of immune signaling during labor. In addition, maternal PB and fetal CB have often been analyzed separately, despite evidence that parturition involves coordinated maternal–fetal immune activation at the maternal–fetal interface. Consistent with this notion, recent transcriptomic studies have revealed reproducible immune activation signatures associated with labor onset and spontaneous PTB (17, 18). However, transcript-level measurements do not necessarily reflect protein abundance or functional signaling activity. Therefore, multiplex immunoassays that enable simultaneous quantification of multiple immune mediators at the protein level in both PB and CB provide an important approach to better characterize immune signaling networks during parturition (19).

In this study, we applied multiplex cytokine profiling to quantify 48 cytokines/chemokines/growth factors in the maternal PB collected at the onset of labor and the paired umbilical CB collected immediately after delivery from term pregnancies, S-PTB and PROM-PTB. We delineated compartment-specific immune mediator landscapes, and found that the immune profile in maternal peripheral circulation remained largely conserved whereas it in fetal circulation exhibited pronounced alterations across labor types and delivery times. Compared with term births, both S-PTB and PROM-PTB were characterized by increased M-SCF and SCF in fetal CB, while additional cytokines/chemokines/growth factors changes further distinguished S-PTB from PROM-PTB. By integrating immune mediator abundance with correlation-based network analyses, we show that fetal, rather than maternal, immune features reflect compartment-specific signaling and subtype-specific immunological heterogeneity in spontaneous PTB, with potential implications for mechanistic stratification and biomarker development.

## 2 Materials and methods

### 2.1 Patients and samples

In this study, a total of 78 women with singleton pregnancies were recruited from the International Peace Maternity and Child Health Hospital (IPMCH) affiliated to Shanghai Jiao Tong University School of Medicine (Shanghai, China) during July 2020 to July 2022, including 26 patients with term births, 26 with S-PTB and 26 with PROM-PTB. All patients were 20–40 years old and delivered their babies by vagina with active labor, which was defined by the presence of regular uterine contractions resulting in cervical change and dilation (20). Maternal PB was collected at the onset of labor, and umbilical CB were collected immediately after delivery. All participants didn’t have: (1) clinical evidence of chorioamnionitis; (2) autoimmune disease, immunodeficiency syndrome, and immunosuppressant or immunomodifying treatment for more than 3 months; (3) virus infections including human immunodeficiency virus, hepatitis B and C virus, and primary herpes simplex virus during current pregnancy; (4) smoking, drinking or other bad habits during current pregnancy; and (5) twin, multiple birth, and iatrogenic PTB. Informed consent was obtained from all participants before enrollment, and this study was approved by the Ethical Committee of the IPMCH hospital with number of (GKLW) 2019-20.

### 2.2 Isolation of human serum

Five milliliters of maternal PB and fetal CB were collected. Samples obtained during daytime were processed immediately, whereas those collected overnight were stored at 4 °C and processed the following morning. Serum was separated within 24 hours of collection by centrifugation at 2000 rpm for 15 minutes at 4 °C, aliquoted to avoid repeated freeze–thaw cycles, and stored at −80 °C until cytokine analysis. All 78 samples collected over the two-year study period were handled using the same standardized protocol.

### 2.3 Multiplex cytokine/chemokine/growth factor assay

The concentrations of 48 cytokines/chemokines/growth factors in the serums were measured using the Bio-Plex Human Cytokine Screening Panel (48-Plex Cat. # 12007283, Bio-Rad) on a Luminex 200 (Luminex, Texas, USA) following the manufacturer’s instructions. During batch testing, all the samples were thawed at room temperature and vortexed properly before use. The 48 cytokines/chemokines/growth factors screening panel includes: Basic fibroblast growth factor (Basic FGF), eotaxin, granulocyte colony-stimulating factor (G-CSF), granulocyte-macrophage colony-stimulating factor (GM-CSF), interferon-gamma (IFN-γ), interleukin-1 beta (IL-1β), interleukin 1 receptor antagonist (IL-1RA), interleukin 1 alpha (IL-1α), interleukin-2 receptor-alpha (IL-2Rα), interleukin-3 (IL-3), interleukin-12 p40 (IL-12 (p40)), interleukin-16 (IL-16), interleukin-2 (IL-2), interleukin-4 (IL-4), interleukin-5 (IL-5), interleukin-6 (IL-6), interleukin-7 (IL-7), interleukin-8 (IL-8), interleukin-9 (IL-9), growth-regulated oncogene-alpha (GRO-α), hepatocyte growth factor (HGF), interferon alpha-2 (IFN-α2), leukemia inhibitory factor (LIF), monocyte-chemotactic protein 3 (MCP-3), interleukin-10 (IL-10), interleukin-12 p70 (IL-12 (p70)), interleukin-13 (IL-13), interleukin-15 (IL-15), interleukin-17A (IL-17A), interferon gamma-inducible protein (IP-10), monocyte chemoattractant protein-1 (MCP-1), monokine induced by gamma interferon (MIG), beta-nerve growth factor (β-NGF), stem cell factor (SCF), stem cell growth factor-beta (SCGF-β), stromal cell-derived factor 1 (SDF-1α), macrophage inflammatory protein-1 alpha (MIP-1α), macrophage inflammatory protein-1 beta (MIP-1β), platelet-derived growth factor-BB (PDGF-BB), regulated upon activation, normal T cell expressed and presumably secreted (RANTES), tumor necrosis factor alpha (TNF-α), vascular endothelial growth factor A (VEGFA), cutaneous T-cell-attracting chemokine (CTACK), macrophage migration inhibitory factor (MIF), TNF-related apoptosis-inducing ligand (TRAIL), interleukin-18 (IL-18), macrophage colony-stimulating factor (M-CSF), and tumor necrosis factor-beta (TNF-β). Raw data were analyzed on a Luminex 200 instrument (Luminex, Texas, USA) fitted with the milliplex analyst version 5.1.0.0 standard and results were reported in pg/ml.

### 2.4 Statistical analysis

**Figure 1:**
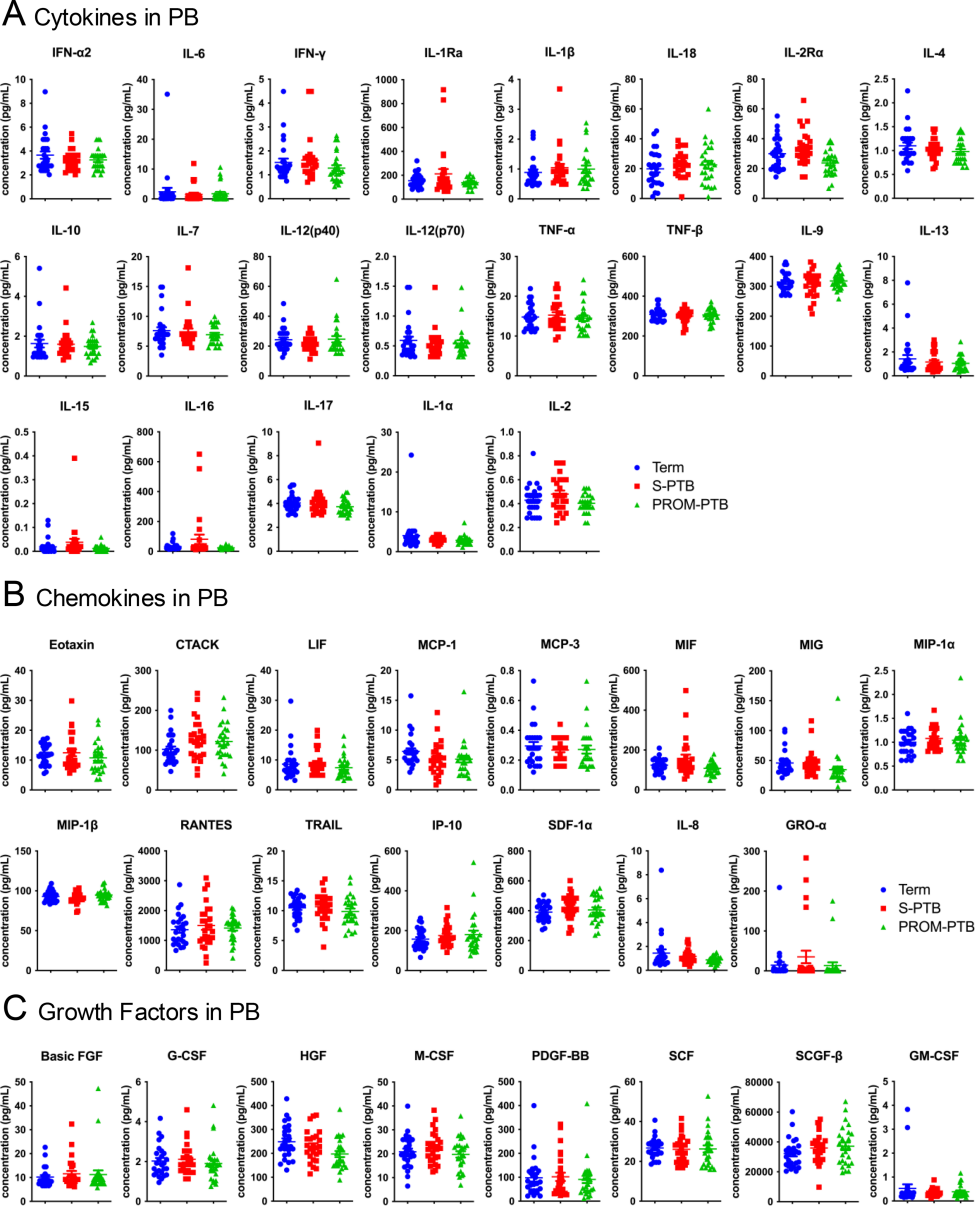
Comparative analysis of immune mediator expression in maternal peripheral blood across term, S-PTB, and PROM-PTB pregnancies. **(A–C)** Expression levels of cytokines **(A)**, chemokines **(B)**, and growth factors **(C)** measured in maternal peripheral blood (PB) of term, S-PTB, and PROM-PTB pregnancies. Data are presented as mean ± standard error of means (SEM). Normality was assessed prior to statistical analysis. In PB, IL-2RA, MIP-1β, SDF-1α, RANTES, IL-18, HGF, TRAIL, and CSF showed normal distributions across the Term, S-PTB and PROM-PTB groups and were analyzed using one-way ANOVA. Variables that did not meet normality assumptions were analyzed using the Kruskal–Wallis test. Raw P values obtained from these group comparisons were subsequently subjected to false discovery rate (FDR) correction to account for multiple testing across the analyzed immune mediators. Only for those with P < 0.05, lines and asterisks are shown.

**Figure 2:**
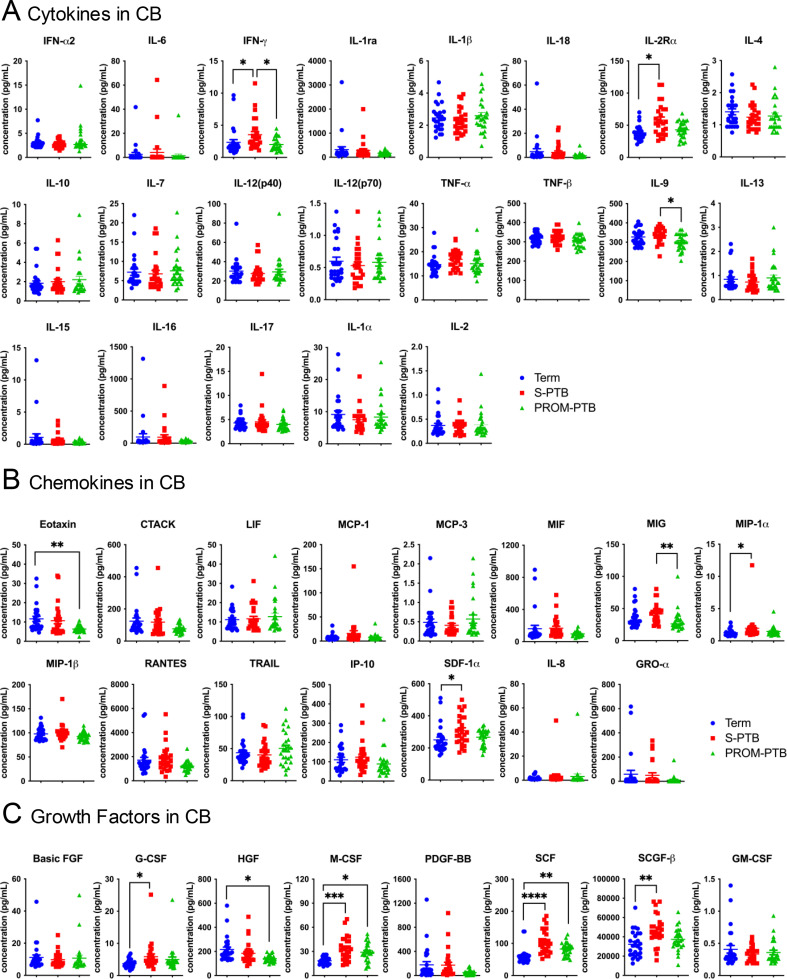
Comparative analysis of immune mediator expression in fetal umbilical cord blood across term, S-PTB, and PROM-PTB pregnancies. **(A–C)** Expression levels of cytokines **(A)**, chemokines **(B)**, and growth factors **(C)** measured in fetal cord blood (CB) from term, S-PTB, and PROM-PTB pregnancies. Data are presented as mean ± SEM. Normality was assessed prior to statistical analysis. In CB, TNF-β, IL-1β, M-CSF, IL-9 and SCGF-β showed normal distributions across the Term, S-PTB, and PROM-PTB groups and were analyzed using one-way ANOVA. Variables that did not meet normality assumptions were analyzed using the Kruskal–Wallis test. Raw P values obtained from these group comparisons were subsequently subjected to false discovery rate (FDR) correction to account for multiple testing across the analyzed immune mediators. Only for those with P < 0.05, lines and asterisks are shown. *P < 0.05; **P < 0.01; ***P < 0.001; ****P < 0.0001.

Clinical characteristics of the study population are summarized in **Table 1**. Fetal gender was compared using the chi-square test, while other clinical parameters were analyzed using one-way ANOVA for normally distributed data or the Kruskal–Wallis test for non-normally distributed data. Cytokine/chemokine/growth factor data were presented as median with interquartile range (IQR) in **Table 2** or mean with standard error of means (SEM) in **Figures 1**, **2**. Data normality was assessed using the Shapiro–Wilk test. For comparisons between two groups, Student’s t-test was applied to normally distributed data, whereas the Mann–Whitney U test was used for non-normally distributed data. Comparisons among three groups were performed using one-way ANOVA for normally distributed data, or the Kruskal–Wallis test for non-normally distributed data. Raw P values obtained from these pairwise and group comparisons were subsequently subjected to false discovery rate (FDR) correction to account for multiple testing across the analyzed immune mediators. The correlation between the two cytokines was assessed using the Pearson correlation coefficient. All tests performed were two-tailed and a p-value <0.05 was considered significant. * P <0.05; ** P <0.01; *** P <0.001; **** P <0.0001.

**Table 1:** Information about maternal demographic characteristics and fetal outcomes.

Characteristics	Term (n = 26)	S-PTB (n = 26)	PROM-PTB (n = 26)	P value
Maternal				
Maternal age (years)	32 (29.25-36)	31.5 (29-34)	30.5 (28.25-34)	0.426
Maternal height (cm)	160 (158-164.75)	160 (158-166.25)	160.5 (156.25-164)	0.928
Prepregnant weight (kg)	52 (48-55)	54 (49-61.25)	55 (51-59.75)	0.315
Prepregnant BMI (kg/m^2^)	19.75 (18.73-21.45)	20.15 (19.58-22.2)	21.8 (19.45-23.4)	0.136
Gestational age (weeks)	39.40 (38.93-39.85)	34.35 (33.93-35)	35 (34.60-35.68)	<0.001
Delivery types, n (%)				
Vaginal delivery	26 (100%)	26 (100%)	26 (100%)	
Cesarean delivery	0 (0%)	0 (0%)	0 (0%)	
Fetal				
Birth weight (g)	3337.5 (3201.25-3438.75)	2277.5 (2126.25-2545)	2277.5 (2126.25-2545)	<0.001
Birth length (cm)	50 (50-50)	46 (43.5-48)	47 (46-48)	<0.001
Placental weight (g)	612.5 (547.5-640)	517.5 (480-566.25)	500 (476.25-561.25)	<0.001
Fetal gender (female)	16/26 (61.54%)	9/26 (34.62%)	14/26 (53.85%)	0.118

Data are given as medians with interquartile range (IQR) or numbers with percentages (%).

**Table 2:** Statistical comparison of the levels of 44 immune mediators including cytokines, chemokines and growth factors in the maternal peripheral blood and fetal cord blood*.

Immune mediators (pg/ml)	PB (n = 78)	CB (n = 78)	P value	Adj. P value
Cytokines				
IFN-α2	3.17 (2.76-3.65)	2.67 (2.38-3.28)	0.016	**0.025**
IL-6	0.46 (0.15-1.39)	0.04 (0.02-0.22)	3.43E-09	**1.37E-08**
IFN-γ	1.26 (1.03-1.59)	1.87 (1.39-3.03)	1.1E-07	**3.72E-07**
IL-1ra	137.7 (103.98-179.61)	163.75 (115.79-231.57)	0.045	0.066
IL-1β	0.80 (0.60-1.06)	2.33 (1.84-2.83)	9.78E-21	**7.17E-20**
IL-18	21.93 (14.06-29.22)	1.00 (0.63-2.13)	1.28E-21	**1.13E-20**
IL-2Rα	28.2 (21.28-34.84)	42.56 (33.73-56.11)	8.02E-11	**3.92E-10**
IL-4	0.99 (0.83-1.24)	1.19 (0.94-1.60)	7.43E-05	**1.63E-04**
IL-10	1.44 (1.14-1.78)	1.49 (1.23-2.11)	0.038	0.058
IL-7	6.98 (5.85-8.27)	5.44 (4.77-8.27)	0.174	0.219
IL-12(p40)	21.24 (18.05-27.55)	27.33 (21.53-33.44)	1.63E-05	**4.22E-05**
IL-12(p70)	0.50 (0.43-0.60)	0.46 (0.34-0.71)	0.998	0.998
TNF-α	14.32 (11.84-17.05)	14.67 (12.40-17.93)	0.404	0.444
TNF-β	307.08 (281.60-320.68)	320.68 (300.50-334.88)	0.011	**0.019**
IL-9	313.88 (294.13-334.95)	327.78 (289.41-347.91)	0.212	0.252
IL-13	0.81 (0.64-1.34)	0.64 (0.53-0.96)	0.015	**0.024**
IL-15	0.01 (0.01-0.02)	0.22 (0.15-0.45)	4.74E-26	**6.95E-25**
IL-16	22.41 (18.44-27.83)	26.08 (20.55-36.49)	0.186	0.227
IL-17A	3.82 (3.29-4.37)	3.89 (3.36-4.76)	0.452	0.485
IL-1α	2.91 (2.35-3.57)	6.25 (5.31-8.66)	5.54E-24	**6.09E-23**
IL-2	0.42 (0.37-0.49)	0.31 (0.23-0.44)	6.95E-06	**2.04E-05**
Chemokines				
Eotaxin	11.26 (7.97-14.29)	7.31 (5.68-10.96)	3.18E-05	**7.77E-05**
CTACK	111.36 (85.88-138.29)	83.18 (65.16-115.67)	0.006	**0.011**
LIF	7.18 (5.42-9.16)	8.92 (7.73-15.59)	1.04E-05	**2.86E-05**
MCP-1	5.34 (4.12-6.35)	6.64 (4.90-8.88)	4.0E-04	**8.38E-04**
MCP-3	0.28 (0.19-0.35)	0.34 (0.22-0.60)	4.49E-05	**1.04E-04**
MIF	121.98 (94.06-147.44)	97.17 (79.09-133.02)	0.058	0.077
MIG	37.74 (30.39-48.68)	32.09 (25.01-43.46)	0.011	**0.019**
MIP-1α	1.00 (0.88-1.21)	1.29 (1.03-1.66)	7.59E-08	**2.78E-07**
MIP-1β	92.86 (87.26-97.77)	93.87 (87.02-103.48)	0.052	0.074
RANTES	1450.00 (1025.00-1724)	1359.00 (1076.75-1724.00)	0.972	0.995
TRAIL	10.82 (8.71-12.05)	38.85 (29.30-53.18)	2.26E-26	**4.97E-25**
IP-10	158.91 (124.87-200.57)	87.575 (60.92-135.47)	1.45E-10	**6.38E-10**
SDF-1α	402.95 (353.82-463.84)	267.00 (212.67-307.35)	4.95E-16	**2.72E-15**
IL-8	1.01 (0.69-1.24)	1.11 (0.76-1.77)	0.003	**0.006**
GRO-α	0.90 (0.20-4.99)	0.40 (0.01-5.24)	0.335	0.378
Growth factors				
Basic FGF	9.21 (7.49-10.62)	7.62 (6.8-11.52)	0.057	0.077
G-CSF	1.84 (1.47-2.25)	4.17 (3.17-5.05)	2.64E-20	**1.66E-19**
HGF	218.83 (168.74-271.75)	153.15 (132.96-195.31)	4.41E-07	**1.39E-06**
M-CSF	20.85 (16.88-24.79)	25.61 (17.25-33.39)	0.004	**0.008**
PDGF-BB	72.25 (43.89-122.82)	63.45 (28.46-104.99)	0.330	0.378
SCF	25.99 (21.01-29.43)	73.08 (57.90-98.96)	9.01E-27	**3.96E-25**
SCGF-β	33564 (26448-40789)	39487 (28082.5-47467)	0.064	0.083
GM-CSF	0.27 (0.23-0.39)	0.29 (0.25-0.38)	0.469	0.491

*All data are presented as median with IQR. Statistical analysis was performed using the Mann–Whitney U test, and P values were adjusted for multiple comparisons using the false discovery rate (FDR) correction (Benjamini–Hochberg method). Bold values indicate statistically significant differences between maternal peripheral blood (PB) and fetal cord blood (CB) after FDR correction.

The limits of detection (LOD) for each cytokine are provided in **Supplementary Data Sheets 1, 2**. Standard curves were generated using a 4-parameter logistic (4-PL) model. To enable continuous statistical analysis, concentrations were recalculated using cubic curve fitting based on signal intensity. Values below the nominal LOD were retained if a fitted value could be obtained; non-fittable measurements were excluded. All analyses were performed using cubic-fitted concentrations (**Supplementary Data Sheets 1, 2**).

## 3 Results

### 3.1 Characteristics of sample cohort

A total of 78 mother–infant dyads were included, comprising term births (Term, n = 26), S-PTB (n = 26) and PROM-PTB (n = 26). The median gestational ages were 39.4 weeks (interquartile range (IQR): 38.93-39.85) in the term group, 34.35 weeks (IQR: 33.93-35) in the S-PTB group, and 35 weeks (IQR: 34.60-35.68) in the PROM-PTB group. All mothers delivered their babies by vagina with active labor, and none of them presented clinical signs of chorioamnionitis. Maternal demographic characteristics and fetal outcomes are summarized in **Table 1**.

As expected, the birth weight and length as well as the placental weight were significantly reduced in the S-PTB and PROM-PTB groups when compared with the term group. Maternal age, and prepregnant height, weight and BMI were comparable among S-PTB, PROM-PTB and term groups.

### 3.2 Immune mediators profiling reveals distinct correlation features in maternal peripheral blood and umbilical cord blood

Maternal PB and fetal CB were collected from the patients, and the multiplex Luminex assay was performed to measure the levels of 48 immune mediators comprising cytokines, chemokines and growth factors in the serum. VEGF, β-NGF, IL-3 and IL-5 were minimally expressed or undetectable in both PB and CB and were therefore excluded from further analysis. Heatmap visualization of the expressions of the remaining 44 immune factors showed that SCGF-β and RANTES were among the most abundantly expressed factors in both PB and CB whereas IL-15, GM-CSF, IL-2, IL-12p70 and MCP-3 displayed consistently low levels (**Supplementary Figure 1**).

**Figure 3:**
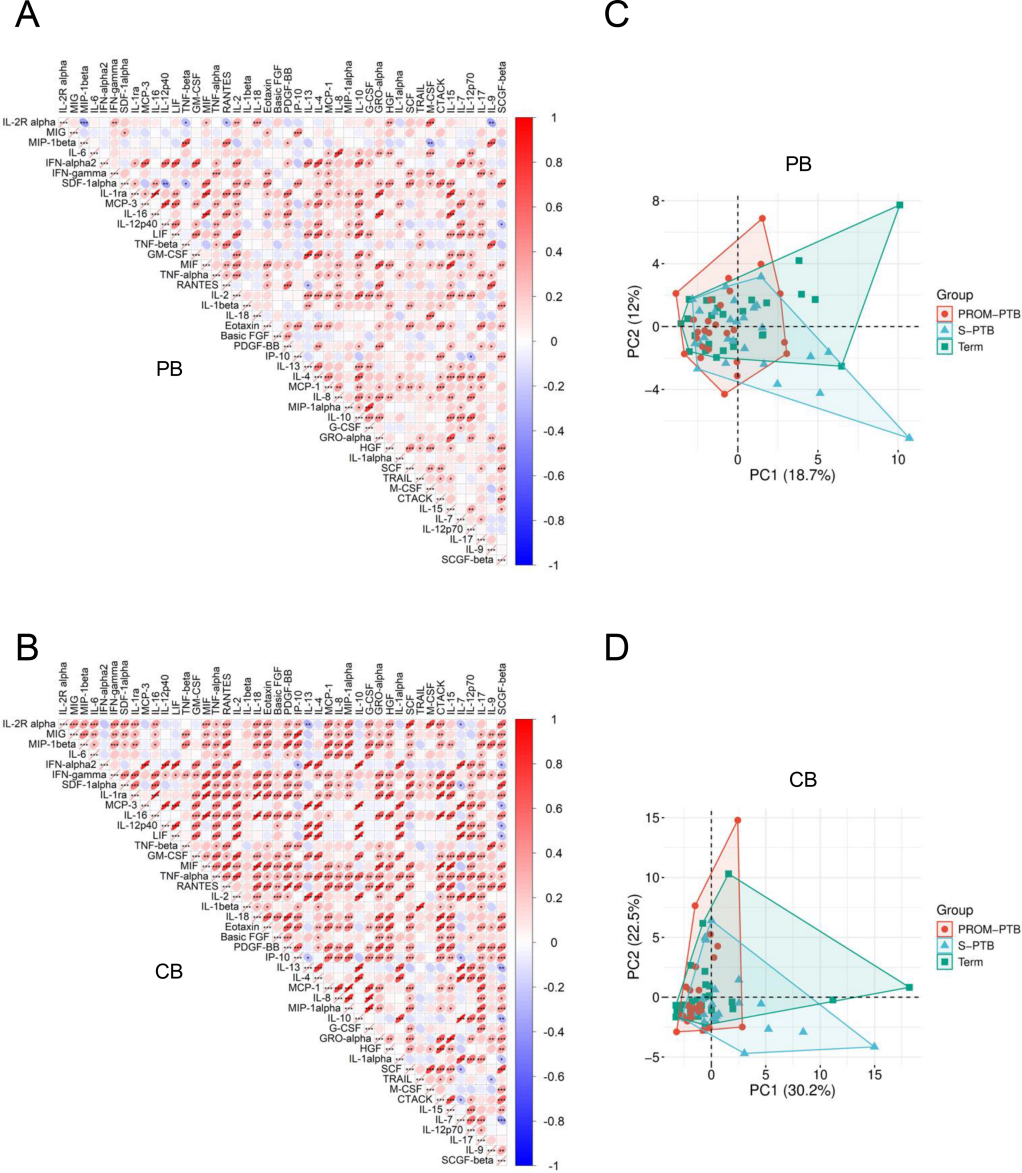
Correlation analysis and principal component analysis of immune mediators in maternal peripheral blood and umbilical cord blood. **(A, B)** Pairwise correlation matrices of immune mediator expression in maternal peripheral blood (PB) **(A)** and fetal cord blood (CB) **(B)**, illustrating distinct correlation architectures between maternal and fetal circulations. Correlation analysis was performed on the original data using Pearson’s correlation. Red indicates positive correlations and blue indicates negative correlations; ellipse shape and color intensity reflect correlation strength. Asterisks denote statistical significance (*P < 0.05; **P < 0.01; ***P < 0.001). Hierarchical clustering was conducted using log_10_-transformed data with Euclidean distance and average linkage. **(C, D)** Principal component analysis (PCA) of immune mediator profiles in maternal PB **(C)** and fetal CB **(D)** from term, S-PTB, and PROM-PTB pregnancies. PCA was performed using the Weishengxin online analysis platform based on all 44 detectable immune mediators in each compartment. PC1 and PC2 represent the major axes of global variance derived from the combined contribution of all measured factors and were used to visualize group separation and overall multivariate structure.

To further characterize the correlations between different immune mediators in the PB and CB, we performed pairwise correlation analyses. In PB, correlations among cytokines, chemokines and growth factors were generally weak to moderate and evenly distributed without clear clustering (**Figure 3A**), suggesting a relatively diffuse correlation structure in the maternal circulation during labor rather than distinct coordinated modules. In contrast, CB displayed a more heterogeneous correlation pattern, with subsets of immune mediators showing stronger interrelationships (**Figure 3B**). Proinflammatory cytokines (e.g., IL-1β, IL-18), lymphocyte-associated factors (e.g., IFN-γ, IL-2Rα, IL-15), chemokines related to leukocyte recruitment (e.g., MCP-1, MCP-3, MIP-1α, IL-8), and hematopoietic growth factors (e.g., G-CSF, M-CSF, SCF) tended to correlate more closely with each other in fetal CB (**Figure 3B**). These correlation features underscore that immune mediator variability is more structured in fetal CB than in maternal PB.

### 3.3 Distinct immune signatures exist in maternal and fetal circulations

Since heatmap analyses revealed a distinct immune mediator signature in the maternal PB and fetal CB (**Supplementary Figure 1**), we next examined the expression of each immune mediator between in maternal and fetal compartment (**Table 2**). Direct comparison showed that maternal PB exhibited significantly higher levels of inflammatory cytokines, including IFN-α2, IL-6, IL-18, IL-13 and IL-2, indicating a more activated maternal inflammatory environment during labor. PB was also enriched for chemokines such as eotaxin, CTACK, MIG, IP-10 and SDF-1α, indicating enhanced chemotactic and antiviral responses in maternal circulation. Among growth factors, HGF was significantly elevated in PB.

In contrast, CB displayed an immune landscape indicative of fetal-specific immunoregulation and hematopoietic activity. CB contained higher concentrations of IFN-γ, IL-1β, IL-2Rα, IL-4, TNF-β, IL-12(p40), IL-15 and IL-1α, reflecting active immune modulation and lymphocyte development within the fetal compartment. CB was also enriched for chemokines including LIF, MCP-1, MCP-3, MIP-1α, TRAIL and IL-8, suggesting a chemokine network supporting fetal leukocyte recruitment and tissue remodeling. Additionally, fetal circulation showed significantly elevated growth factors such as G-CSF, M-CSF and SCF, indicating a heightened hematopoietic demand for fetal development at late gestation.

Importantly, paired analyses within each group further revealed that while several maternal–fetal gradients (e.g., higher IL-1β, IL-2Rα, IL-12p40, IL-15, G-CSF, SCF, and TRAIL in CB and higher IL-6, IL-18, IP-10, and SDF-1α in PB) were preserved across term and preterm pregnancies, distinct alterations emerged in PTB subtypes (**Supplementary Tables 1**–**4**). In both S-PTB and PROM-PTB, IL-2 and HGF were reduced in CB relative to PB, whereas IFN-γ, MCP-1, MIP-1α, and M-CSF were increased in CB, and these patterns were not observed in term deliveries (**Supplementary Tables 1**–**4**). Moreover, S-PTB and PROM-PTB exhibited partially divergent CB–PB gradients for selected mediators, indicating phenotype-specific maternal–fetal immune dysregulation.

Together, these findings demonstrate that maternal PB is dominated by inflammatory and chemotactic signals, whereas fetal CB exhibits a profile enriched in regulatory cytokines and hematopoietic growth factors. Furthermore, PTB is associated with selective disruption of the physiological maternal–fetal immune balance, underscoring both shared and subtype-specific immune alterations in S-PTB and PROM-PTB.

### 3.4 Maternal peripheral blood immune profiles are largely conserved across labor types and delivery times

To assess whether systemic immune signatures differ among term, S-PTB and PROM-PTB pregnancies, principal component analysis (PCA) was performed for the expressions of immune mediators in the maternal PB and fetal CB (**Figures 3C, D**). PCA data demonstrated substantial overlap among term, S-PTB and PROM-PTB samples in both PB and CB, with only modest shifts of S-PTB and PROM-PTB away from term pregnancies, indicating subtle immune deviations rather than broad immune-mediators remodeling in the maternal and fetal circulation.

Next, we compared the expression of each immune mediator in the maternal PB across term, S-PTB and PROM-PTB pregnancy. Overall, cytokine, chemokine and growth factor levels were largely conserved, with no significant group-specific variation in maternal PB (**Figures 1A–C**). Taken together, these findings demonstrate that maternal systemic immunity remains largely stable across labor types and delivery times, supporting the view that PTB–associated immune alterations are unlikely to originate from maternal peripheral circulation.

### 3.5 Cord blood displays a marked difference in immune mediators across labor types and delivery times

Unlike the conserved immune profiling observed in maternal circulation, fetal CB showed more group-specific differences across term, S-PTB and PROM-PTB pregnancies (**Figures 2A–C**). Compared with term deliveries, S-PTB was characterized by coordinated upregulation of multiple immune mediators, including the cytokine IFN-γ and IL-2Rα, and the chemokines MIP-1α and SDF-1α, together with elevated levels of the hematopoietic growth factors G-CSF, M-CSF, SCF and SCGF-β. This pattern suggests enhanced immune activation and growth factor–driven hematopoietic signaling in the fetal compartment during spontaneous preterm labor.

In contrast, PROM-PTB displayed a distinct immune profile. Relative to term pregnancies, PROM-PTB was associated with reduced levels of the chemokine eotaxin, as well as the growth factor HGF, alongside increased M-CSF and SCF. These changes indicate attenuated chemokine signaling coupled with selective activation of growth factor pathways in the fetal circulation of PROM-PTB.

Taken together, these results show that fetal immune profiles differ between S-PTB and PROM-PTB. Both preterm groups exhibited increased levels of growth factors, including M-CSF and SCF, relative to term births. In addition, S-PTB was associated with broader changes in cytokine, chemokine and growth factor expression, whereas PROM-PTB displayed more limited alterations. These findings indicate that fetal immune responses vary across PTB subtypes, and are more prominently altered than those observed in maternal peripheral circulation.

## 4 Discussion

Spontaneous PTB is widely recognized as a multifactorial syndrome involving immune activation, yet the relative contributions of maternal versus fetal immune dynamics remain incompletely understood. In this study, comprehensive multiplex profiling of paired maternal PB and fetal CB revealed that maternal systemic immune mediator levels were largely conserved across term, S-PTB and PROM-PTB pregnancies, with only modest variation in a limited subset of analytes. In contrast, fetal CB exhibited pronounced subtype-associated immune alterations, particularly in hematopoietic growth factors: both S-PTB and PROM-PTB were characterized by elevated M-CSF and SCF relative to term births, while SCGF-β was selectively elevated in S-PTB and HGF was only reduced in PROM-PTB. Direct comparison further revealed lower IFN-γ and IL-9 in PROM-PTB versus S-PTB (**Figures 2A–C**). These observations highlight growth factor network remodeling in fetal circulation that differentiates spontaneous PTB subtypes and cannot be captured by maternal systemic profiling alone.

Compared with previous studies reporting elevated inflammatory mediators in maternal circulation during PTB, our findings revealed a distinct pattern. While earlier reports described increased cytokines and chemokines such as CXCL1, IL-1β, TNF-α, and IL-6 in maternal PB (8, 11, 21), our analysis—after correction for multiple comparisons—identified largely stable cytokine and chemokine levels in maternal PB across term, S-PTB, and PROM-PTB pregnancies. In fetal circulation, prior studies have reported associations between PTB and altered CB levels of inflammatory mediators, including elevated IL-6, MCP-1, IL-15, IL-8, MCP-1, MIP-1α and TNF-α, as well as reduced IL-18, IL-1β and eotaxin (10, 14–16). In our cohort, we observed subtype-specific immune alterations: MIP-1α was increased in S-PTB compared with term, whereas eotaxin was decreased in PROM-PTB, consistent with findings reported in previous studies. Additional subtype-associated changes were also detected in hematopoietic growth factors, including M-CSF, SCF and SCGF-β. These differences may reflect variations in timing of sample collection, biomarker panels, cohort composition, analytical methods, and statistical correction strategies across studies. Extending these findings, our simultaneous profiling of maternal and fetal compartments across clinically defined PTB subtypes demonstrates that immune alterations related to spontaneous PTB are more prominently structured within the fetal circulation than in maternal compartment. These results highlight the value of a paired, subtype-aware maternal–fetal immune framework for capturing coordinated yet compartment-specific immune dynamics underlying PTB.

Among the immune mediators that distinguished PTB subtypes in fetal circulation, colony-stimulating factors such as M-CSF and SCF are biologically plausible contributors to PTB-related processes. M-CSF (also known as CSF-1) is a hematopoietic growth factor that regulates monocyte/macrophage differentiation (22). M-CSF has been implicated in trophoblast differentiation, placental growth, and implantation (23, 24). Maternal serum M-CSF levels normally increase throughout pregnancy, whereas insufficient elevation has been associated with adverse obstetrics outcomes such as hypertensive disorders and preeclampsia (13). In this context, our observation of elevated M-CSF levels in the CB of both S-PTB and PROM-PTB suggests that dysregulated M-CSF signaling may reflect altered fetal immune responses in pathological pregnancies, potentially contributing to preterm parturition. SCF (also known as KITLG) is highly expressed in decidual and placental tissues, particularly within the placental villous core, whereas its receptor Kit is mainly localized to trophoblasts, supporting a paracrine SCF–Kit signaling axis regulating trophoblast survival and proliferation (25). In this context, elevated SCF levels in CB from preterm infants may reflect dysregulated trophoblast functions associated with pathological pregnancies including PTB. SCGF-β has not been previously linked to PTB; however, in our study, it was elevated in the CB of S-PTB but unchanged in PROM-PTB, suggesting subtype-specific roles of SCGF-β that warrant further investigation. Similarly, while decreased HGF has been reported in preterm CB (26), we observed this reduction only in PROM-PTB, with levels remaining stable in S-PTB without membrane rupture, highlighting distinct underlying mechanisms that require further exploration.

Nevertheless, our study provides a compartment-resolved framework showing that fetal immune and hematopoietic growth factor signatures carry PTB-specific information that maternal systemic profiling at delivery does not capture. This refined view highlights the heterogeneity of spontaneous PTB and its potential for mechanistic stratification and biomarker development. However, since maternal PB was collected at the onset of labor, the observed immune alterations likely represent consequential changes associated with parturition rather than early predictive signals. Consequently, the cross-sectional design at labor onset limits the assessment of temporal immune trajectories and the calculation of receiver operating characteristic (ROC) curves to evaluate these factors as early predictive biomarkers. Furthermore, soluble mediator profiling cannot resolve cellular sources or functional pathways. In addition, fetal sex may also influence the risk or immunological features of PTB; therefore, larger cohorts and more robust statistical approaches will be required to determine its independent contribution. Future studies integrating longitudinal sampling prior to labor onset, single-cell phenotyping, and placental or membrane tissue analysis will be essential to elucidate causal mechanisms and identify early predictive markers or therapeutic targets for spontaneous PTB.

## Data Availability

The original contributions presented in the study are included in the article/**Supplementary Material**. Further inquiries can be directed to the corresponding authors.

## Supplementary material

Supplementary Figure 1 Distinct cytokine expression patterns in maternal peripheral blood and umbilical cord blood. Heatmap showing the mean expression levels of 44 detectable immune mediators in maternal peripheral blood (PB) and umbilical cord blood (CB) of 78 mother–infant dyads. Values were averaged within each group and visualized using the Weishengxin online analysis platform based on the original (non-transformed) data. Color gradients (blue to red) indicate relatively lower to higher expression levels.

## References

[1] Ohuma EO Moller AB Bradley E Chakwera S Hussain-Alkhateeb L Lewin A . National, regional, and global estimates of preterm birth in 2020, with trends from 2010: a systematic analysis. Lancet. (2023) 402:1261–71. doi: 10.1016/s0140-6736(23)00878-4, PMID: 37805217

[2] Ferreira A Bernardes J Gonçalves H . Risk scoring systems for preterm birth and their performance: A systematic review. J Clin Med. (2023) 12. doi: 10.3390/jcm12134360, PMID: 37445395 PMC10342801

[3] Romero R Dey SK Fisher SJ . Preterm labor: one syndrome, many causes. Science. (2014) 345:760–5. doi: 10.1126/science.1251816, PMID: 25124429 PMC4191866

[4] Goldenberg RL Culhane JF Iams JD Romero R . Epidemiology and causes of preterm birth. Lancet. (2008) 371:75–84. doi: 10.1016/s0140-6736(08)60074-4, PMID: 18177778 PMC7134569

[5] Lv M Jia Y Dong J Wu S Ying H . The landscape of decidual immune cells at the maternal-fetal interface in parturition and preterm birth. Inflammation Res. (2025) 74:44. doi: 10.1007/s00011-025-02015-6, PMID: 40038160 PMC11880140

[6] Hamburg-Shields E Mesiano S . The hormonal control of parturition. Physiol Rev. (2024) 104:1121–45. doi: 10.1152/physrev.00019.2023, PMID: 38329421 PMC11380996

[7] Garcia-Flores V Romero R Tarca AL Peyvandipour A Xu Y Galaz J . Deciphering maternal-fetal cross-talk in the human placenta during parturition using single-cell RNA sequencing. Sci Transl Med. (2024) 16:eadh8335. doi: 10.1126/scitranslmed.adh8335, PMID: 38198568 PMC11238316

[8] Svenvik M Jenmalm MC Brudin L Raffetseder J Hellberg S Axelsson D . Chemokine and cytokine profiles in preterm and term labor, in preterm prelabor rupture of the membranes, and in normal pregnancy. J Reprod Immunol. (2024) 164:104278. doi: 10.1016/j.jri.2024.104278, PMID: 38901109

[9] Gomez-Lopez N StLouis D Lehr MA Sanchez-Rodriguez EN Arenas-Hernandez M . Immune cells in term and preterm labor. Cell Mol Immunol. (2014) 11:571–81. doi: 10.1038/cmi.2014.46, PMID: 24954221 PMC4220837

[10] Otsubo Y Hashimoto K Kanbe T Sumi M Moriuchi H . Association of cord blood chemokines and other biomarkers with neonatal complications following intrauterine inflammation. PLoS One. (2017) 12:e0175082. doi: 10.1371/journal.pone.0175082, PMID: 28531215 PMC5439663

[11] Laudanski P Lemancewicz A Kuc P Charkiewicz K Ramotowska B Kretowska M . Chemokines profiling of patients with preterm birth. Mediators Inflammation. (2014) 2014:185758. doi: 10.1155/2014/185758, PMID: 24876667 PMC4020160

[12] Hoffmann JA Gründler K Richter DU Stubert J . Prediction of spontaneous preterm birth using CCL2 and CXCL10 in maternal serum of symptomatic high-risk pregnant women: a prospective cohort study. BMC Pregnancy Childbirth. (2023) 23:697. doi: 10.1186/s12884-023-06016-3, PMID: 37770883 PMC10537471

[13] keith JC Jr. Pijnenborg R Luyten C Spitz B Schaub R Van Assche FA . Maternal serum levels of macrophage colony-stimulating factor are associated with adverse pregnancy outcome. Eur J Obstet Gynecol Reprod Biol. (2000) 89:19–25. doi: 10.1016/s0301-2115(99)00154-2, PMID: 10733019

[14] Neta GI von Ehrenstein OS Goldman LR Lum K Sundaram R Andrews W . Umbilical cord serum cytokine levels and risks of small-for-gestational-age and preterm birth. Am J Epidemiol. (2010) 171:859–67. doi: 10.1093/aje/kwq028, PMID: 20348155 PMC2877445

[15] Anderson J Thang CM Thanh LQ Dai VTT Phan VT Nhu BTH . Immune profiling of cord blood from preterm and term infants reveals distinct differences in pro-inflammatory responses. Front Immunol. (2021) 12:777927. doi: 10.3389/fimmu.2021.777927, PMID: 34790206 PMC8591285

[16] Sullivan G Galdi P Borbye-Lorenzen N Stoye DQ Lamb GJ Evans MJ . Preterm birth is associated with immune dysregulation which persists in infants exposed to histologic chorioamnionitis. Front Immunol. (2021) 12:722489. doi: 10.3389/fimmu.2021.722489, PMID: 34512648 PMC8430209

[17] Gomez-Lopez N Romero R Galaz J Bhatti G Done B Miller D . Transcriptome changes in maternal peripheral blood during term parturition mimic perturbations preceding spontaneous preterm birth†. Biol Reprod. (2022) 106:185–99. doi: 10.1093/biolre/ioab197, PMID: 34686873 PMC8897989

[18] Pereyra S Sosa C Bertoni B Sapiro R . Transcriptomic analysis of fetal membranes reveals pathways involved in preterm birth. BMC Med Genomics. (2019) 12:53. doi: 10.1186/s12920-019-0498-3, PMID: 30935390 PMC6444860

[19] Martínez VP Di Paola N Alonso DO Pérez-Sautu U Bellomo CM Iglesias AA . Super-spreaders" and person-to-person transmission of andes virus in Argentina. N Engl J Med. (2020) 383:2230–41. doi: 10.1056/NEJMoa2009040, PMID: 33264545

[20] Jiang Y Lai X Liu Y Yang C Liu Z Liu X . CD8(+) T cells in fetal membranes display a unique phenotype, and their activation is involved in the pathophysiology of spontaneous preterm birth. J Pathol. (2024) 262:240–53. doi: 10.1002/path.6229, PMID: 38018407

[21] Lyon D Cheng CY Howland L Rattican D Jallo N Pickler R . Integrated review of cytokines in maternal, cord, and newborn blood: part I–associations with preterm birth. Biol Res Nurs. (2010) 11:371–6. doi: 10.1177/1099800409344620, PMID: 20034950

[22] Praloran V . Structure, biosynthesis and biological roles of monocyte-macrophage colony stimulating factor (CSF-1 or M-CSF). Nouv Rev Fr Hematol (1978). (1991) 33:323–33. 1838150

[23] Eckmann-Scholz C Wilke C Acil Y Alkatout I Salmassi A . Macrophage colony-stimulating factor (M-CSF) in first trimester maternal serum: correlation with pathologic pregnancy outcome. Arch Gynecol Obstet. (2016) 293:1213–7. doi: 10.1007/s00404-015-3931-7, PMID: 26538356

[24] Saito S Saito M Enomoto M Ito A Motoyoshi K Nakagawa T . Human macrophage colony-stimulating factor induces the differentiation of trophoblast. Growth Factors. (1993) 9:11–9. doi: 10.3109/08977199308991578, PMID: 8347348

[25] Kauma S Huff T Krystal G Ryan J Takacs P Turner T . The expression of stem cell factor and its receptor, c-kit in human endometrium and placental tissues during pregnancy. J Clin Endocrinol Metab. (1996) 81:1261–6. doi: 10.1210/jcem.81.3.8772609, PMID: 8772609

[26] Khan N Couper J Goldsworthy W Aldis J McPhee A Couper R . Relationship of hepatocyte growth factor in human umbilical vein serum to gestational age in normal pregnancies. Pediatr Res. (1996) 39:386–9. doi: 10.1203/00006450-199603000-00002, PMID: 8929855

