# Supplementary figures and images for: Cytokine profiling of maternal peripheral and umbilical cord blood in term and preterm labor
Source: Front Immunol. 2026 Apr 7;17:1786565. doi: 10.3389/fimmu.2026.1786565 (PMC13095561; doi:10.3389/fimmu.2026.1786565)

Supplementary Figure 1

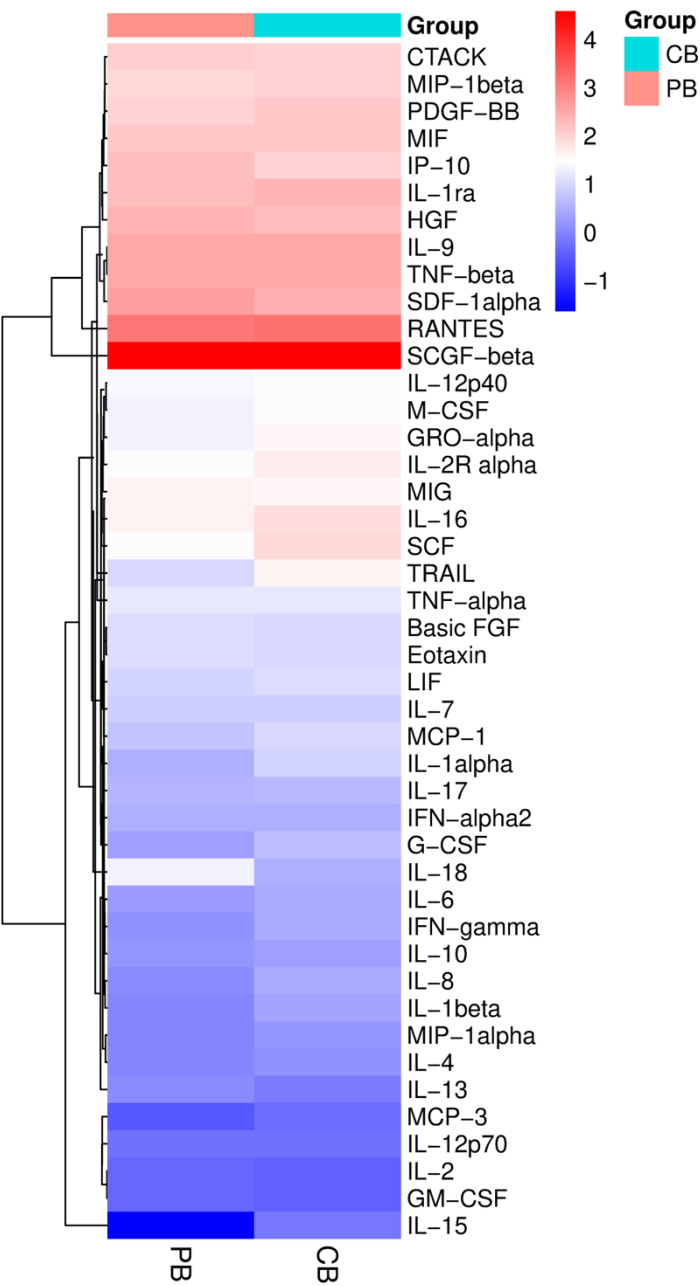

Supplement: Supplementary Figure 1 — Distinct cytokine expression patterns in maternal peripheral blood and umbilical cord blood. Heatmap showing the mean expression levels of 44 detectable immune mediators in maternal peripheral blood (PB) and umbilical cord blood (CB) of 78 mother–infant dyads. Values were averaged within each group and visualized using the Weishengxin online analysis platform based on the original (non-transformed) data. Color gradients (blue to red) indicate relatively lower to higher expression levels. [file Image1.pdf]
