# Supplementary material for: Cytokine profiling of maternal peripheral and umbilical cord blood in term and preterm labor
Source: Front Immunol. 2026 Apr 7;17:1786565. doi: 10.3389/fimmu.2026.1786565 (PMC13095561; doi:10.3389/fimmu.2026.1786565)
Supplement: Supplementary file 4 [file Table1.docx]

**Supplementary Table 1. Statistical comparison of the levels of 44 immune mediators in the maternal peripheral blood and fetal cord blood from term births.**

| **Immune mediators (pg/ml)** | **PB**  **(n = 26)** | **CB**  **(n = 26)** | **P value** | **Adj. P value** | **Trend** |
| --- | --- | --- | --- | --- | --- |
| **Cytokines** |  |  |  |  |  |
| IFN-α2 | 3.07 (2.76–4.21) | 2.82 (2.38–3.28) | 0.119 | 0.218 |  |
| IL-6 | 0.45 (0.14–1.56) | 0.12 (0.02–0.46) | 0.009 | **0.038** | ↓ |
| IFN-γ | 1.26 (1.14–1.51) | 1.65 (1.30–1.94) | 0.031 | 0.071 |  |
| IL-1ra | 137.70 (106.49–190.56) | 163.75 (131.86–203.35) | 0.283 | 0.415 |  |
| IL-1β | 0.76 (0.55–0.92) | 2.28 (1.77–2.81) | 1.04e-07 | **9.15E-07** | ↑ |
| IL-18 | 18.31 (10.50–29.22) | 1.20 (0.70–2.98) | 4.15e-07 | **3.04E-06** | ↓ |
| IL-2Rα | 29.46 (20.61–36.19) | 35.41 (30.93–42.82) | 0.011 | **0.042** | ↑ |
| IL-4 | 0.99 (0.91–1.25) | 1.24 (1.08–1.60) | 0.013 | **0.044** | ↑ |
| IL-10 | 1.24 (1.07–1.98) | 1.49 (1.23–1.78) | 0.435 | 0.563 |  |
| IL-7 | 6.98 (5.85–8.10) | 5.85 (4.77–8.27) | 0.180 | 0.280 |  |
| IL-12p40 | 21.24 (18.44–28.49) | 27.33 (24.35–33.92) | 0.015 | **0.047** | ↑ |
| IL-12p70 | 0.50 (0.41–0.64) | 0.44 (0.35–0.79) | 0.687 | 0.795 |  |
| TNF-α | 14.15 (11.84–16.84) | 14.32 (11.79–15.71) | 0.804 | 0.885 |  |
| TNF-β | 307.08 (277.07–320.68) | 320.68 (300.50–334.88) | 0.144 | 0.245 |  |
| IL-9 | 307.15 (294.13–338.66) | 324.27 (294.13–349.78) | 0.312 | 0.443 |  |
| IL-13 | 0.73 (0.64–1.32) | 0.64 (0.57–0.85) | 0.157 | 0.255 |  |
| IL-15 | 0.01 (0.00–0.01) | 0.23 (0.18–0.49) | 1.14e-09 | **1.67E-08** | ↑ |
| IL-16 | 22.91 (18.65–33.19) | 23.91 (20.55–35.76) | 0.602 | 0.730 |  |
| IL-17 | 3.89 (3.55–4.37) | 4.17 (3.36–4.99) | 0.614 | 0.730 |  |
| IL-2 | 0.42 (0.37–0.47) | 0.30 (0.24–0.44) | 0.027 | 0.066 |  |
| **Chemokines** |  |  |  |  |  |
| Eotaxin | 11.64 (9.17–14.60) | 9.20 (6.86–14.30) | 0.184 | 0.280 |  |
| CTACK | 91.64 (74.29–121.53) | 90.71 (69.93–124.19) | 1.000 | 1.000 |  |
| LIF | 7.18 (5.97–9.59) | 8.92 (6.77–14.93) | 0.013 | **0.044** | ↑ |
| MCP-1 | 6.09 (4.90–6.63) | 6.35 (4.47–8.14) | 0.862 | 0.925 |  |
| MCP-3 | 0.28 (0.20–0.35) | 0.38 (0.22–0.60) | 0.045 | 0.099 |  |
| MIF | 123.32 (95.64–149.87) | 84.42 (72.93–110.09) | 0.018 | 0.053 |  |
| MIG | 42.52 (32.27–50.57) | 30.39 (27.87–40.51) | 0.023 | 0.060 |  |
| MIP-1α | 0.99 (0.81–1.21) | 1.13 (0.94–1.42) | 0.068 | 0.143 |  |
| MIP-1β | 92.36 (87.02–98.05) | 92.36 (87.02–106.61) | 0.335 | 0.461 |  |
| RANTES | 1316.00 (930.17–1616.00) | 1419.00 (1124.00–1688.25) | 0.498 | 0.626 |  |
| IP-10 | 139.54 (118.29–196.41) | 81.05 (59.95–159.83) | 0.003 | **0.014** | ↓ |
| SDF-1α | 385.86 (345.34–444.17) | 224.52 (196.09–267.03) | 8.83e-07 | **5.55E-06** | ↓ |
| IL-8 | 1.07 (0.66–1.47) | 1.25 (0.93–1.77) | 0.117 | 0.218 |  |
| GRO-α | 0.64 (0.30–6.00) | 0.60 (0.02–10.64) | 0.395 | 0.526 |  |
| **Growth Factors** |  |  |  |  |  |
| Basic FGF | 8.53 (7.49–10.62) | 9.85 (7.31–11.90) | 0.971 | 0.993 |  |
| G-CSF | 1.88 (1.45–2.41) | 3.46 (2.86–4.33) | 2.21e-06 | **1.22E-05** | ↑ |
| HGF | 231.02 (200.81–294.76) | 182.04 (145.79–240.02) | 0.022 | 0.060 |  |
| M-CSF | 20.18 (17.35–25.62) | 18.12 (14.28–20.85) | 0.113 | 0.218 |  |
| PDGF-BB | 77.15 (43.89–126.17) | 80.49 (36.91–175.55) | 0.940 | 0.985 |  |
| SCF | 26.99 (23.78–29.27) | 55.75 (50.57–61.45) | 8.05e-10 | **1.67E-08** | ↑ |
| SCGF-β | 31796.50 (25058.25–35818.00) | 30448.50 (21764.00–42842.50) | 0.749 | 0.845 |  |
| GM-CSF | 0.27 (0.20–0.38) | 0.29 (0.26–0.46) | 0.145 | 0.245 |  |
| TRAIL | 10.82 (9.19–11.92) | 38.85 (32.31–46.19) | 6.45e-10 | **1.67E-08** | ↑ |

All data are presented as median with IQR. Bold values represent significantly different cytokines, chemokines and growth factors between maternal peripheral blood (PB) and fetal cord blood (CB). Statistical analysis was performed using the Mann–Whitney U test, and P values were adjusted for multiple comparisons using the false discovery rate (FDR) correction (Benjamini–Hochberg method).

**Supplementary Table 2. Statistical comparison of the levels of 44 immune mediators in the maternal peripheral blood and fetal cord blood from S-PTB group.**

| **Immune mediators (pg/ml)** | **PB**  **(n = 26)** | **CB**  **(n = 26)** | **P value** | **Adj. P value** | **Trend** |
| --- | --- | --- | --- | --- | --- |
| **Cytokines** |  |  |  |  |  |
| IFN-α2 | 3.17 (2.76–3.65) | 2.60 (2.12–3.08) | 0.021 | **0.038** | ↓ |
| IL-6 | 0.43 (0.15–0.71) | 0.04 (0.02–0.17) | 7.00E-04 | **0.002** | ↓ |
| IFN-γ | 1.40 (1.14–1.67) | 2.66 (1.92–4.29) | 1.36E-05 | **7.48E-05** | ↑ |
| IL-1ra | 137.70 (101.68–195.26) | 169.25 (108.58–256.67) | 0.464 | 0.567 |  |
| IL-1β | 0.92 (0.62–1.06) | 2.13 (1.77–2.63) | 9.76E-08 | **6.13E-07** | ↑ |
| IL-18 | 23.15 (17.30–28.13) | 1.50 (0.73–3.98) | 6.01E-08 | **4.41E-07** | ↓ |
| IL-2Rα | 32.13 (27.31–38.00) | 54.03 (36.82–73.96) | 4.10E-05 | **1.80E-04** | ↑ |
| IL-4 | 1.03 (0.94–1.13) | 1.16 (0.96–1.43) | 0.056 | 0.088 |  |
| IL-10 | 1.50 (1.19–1.78) | 1.55 (1.23–1.91) | 0.515 | 0.612 |  |
| IL-7 | 6.98 (6.25–7.95) | 5.27 (4.16–7.42) | 0.011 | **0.024** | ↓ |
| IL-12p40 | 21.24 (18.05–24.72) | 25.84 (21.53–29.69) | 0.010 | **0.021** | ↑ |
| IL-12p70 | 0.50 (0.43–0.60) | 0.50 (0.34–0.69) | 0.819 | 0.857 |  |
| TNF-α | 14.15 (12.69–17.37) | 17.49 (13.65–19.34) | 0.138 | 0.195 |  |
| TNF-β | 307.08 (283.14–320.68) | 327.70 (307.92–346.91) | 0.015 | **0.029** | ↑ |
| IL-9 | 313.88 (277.14–334.95) | 338.62 (315.60–357.44) | 0.014 | **0.028** | ↑ |
| IL-13 | 0.81 (0.64–1.70) | 0.57 (0.47–0.96) | 0.039 | 0.069 |  |
| IL-15 | 0.02 (0.01–0.03) | 0.35 (0.16–0.55) | 3.66E-09 | **4.03E-08** | ↑ |
| IL-16 | 23.66 (18.86–29.72) | 26.66 (19.38–63.43) | 0.442 | 0.556 |  |
| IL-17 | 3.96 (3.55–4.37) | 3.89 (3.52–4.69) | 0.667 | 0.752 |  |
| IL-2 | 0.47 (0.37–0.60) | 0.37 (0.23–0.44) | 0.004 | **0.010** | ↓ |
| **Chemokines** |  |  |  |  |  |
| Eotaxin | 10.11 (8.32–15.55) | 8.15 (5.41–12.49) | 0.047 | 0.079 |  |
| CTACK | 122.77 (88.24–146.02) | 98.87 (62.40–151.22) | 0.253 | 0.337 |  |
| LIF | 8.20 (6.27–8.55) | 9.59 (7.73–14.45) | 0.052 | 0.084 |  |
| MCP-1 | 4.90 (3.58–6.56) | 7.88 (5.20–12.37) | 0.002 | **0.006** | ↑ |
| MCP-3 | 0.28 (0.21–0.35) | 0.28 (0.22–0.58) | 0.09 | 0.136 |  |
| MIF | 134.48 (107.70–159.94) | 127.50 (90.56–211.95) | 0.742 | 0.796 |  |
| MIG | 42.07 (33.56–50.57) | 42.06 (31.91–47.64) | 0.589 | 0.682 |  |
| MIP-1α | 1.01 (0.88–1.28) | 1.49 (1.19–1.94) | 2.14E-05 | **1.05E-04** | ↑ |
| MIP-1β | 88.92 (87.50–96.97) | 98.04 (92.86–103.48) | 0.009 | **0.020** | ↑ |
| RANTES | 1406.00 (998.32–1880.00) | 1549.00 (1206.25–2086.50) | 0.318 | 0.412 |  |
| IP-10 | 161.51 (138.09–200.57) | 100.27 (75.87–148.20) | 3.00E-04 | **0.001** | ↓ |
| SDF-1α | 416.23 (380.69–481.78) | 288.88 (243.52–383.91) | 7.10E-05 | **2.84E-04** | ↓ |
| IL-8 | 1.07 (0.84–1.37) | 1.31 (0.84–2.61) | 0.115 | 0.169 |  |
| GRO-α | 2.11 (0.17–7.27) | 0.78 (0.12–10.61) | 0.742 | 0.796 |  |
| **Growth Factors** |  |  |  |  |  |
| Basic FGF | 9.21 (8.37–13.50) | 7.62 (6.80–12.44) | 0.18 | 0.248 |  |
| G-CSF | 1.84 (1.57–2.46) | 4.60 (3.83–6.15) | 2.28E-08 | **2.01E-07** | ↑ |
| HGF | 226.07 (190.12–264.51) | 165.16 (130.82–203.96) | 0.003 | **0.008** | ↓ |
| M-CSF | 22.25 (17.73–25.62) | 32.85 (23.81–42.60) | 0.002 | **0.005** | ↑ |
| PDGF-BB | 66.38 (37.75–132.83) | 80.49 (29.11–202.13) | 0.84 | 0.860 |  |
| SCF | 25.57 (20.64–29.91) | 96.86 (73.48–123.82) | 6.48E-10 | **1.43E-08** | ↑ |
| SCGF-β | 36610.50  (28862.50–40789.00) | 44967.00  (40133.50–52335.50) | 0.002 | **0.007** | ↑ |
| GM-CSF | 0.32 (0.23–0.39) | 0.34 (0.22–0.38) | 0.905 | 0.905 |  |
| TRAIL | 10.94 (9.61–12.05) | 34.11 (27.17–48.77) | 6.44E-10 | **1.43E-08** | ↑ |

All data are presented as median with IQR. Bold values represent significantly different cytokines, chemokines and growth factors between maternal peripheral blood (PB) and fetal cord blood (CB). Statistical analysis was performed using the Mann–Whitney U test, and P values were adjusted for multiple comparisons using the false discovery rate (FDR) correction (Benjamini–Hochberg method).

**Supplementary Table 3. Statistical comparison of the levels of 44 immune mediators in the maternal peripheral blood and fetal cord blood from PROM-PTB group.**

| **Immune mediators (pg/ml)** | **PB**  **(n = 26)** | **CB**  **(n = 26)** | **P value** | **Adj. P value** | **Trend** |
| --- | --- | --- | --- | --- | --- |
| **Cytokines** |  |  |  |  |  |
| IFN-α2 | 3.17 (2.76–3.65) | 2.67 (2.38–3.52) | 0.334 | 0.433 |  |
| IL-6 | 0.46 (0.14–1.56) | 0.04 (0.02–0.09) | 1.00E-04 | **3.39E-04** | ↓ |
| IFN-γ | 1.11 (0.83–1.51) | 1.52 (1.32–2.76) | 0.002 | **0.004** | ↑ |
| IL-1ra | 137.70 (120.92–156.81) | 148.62 (115.79–174.74) | 0.291 | 0.400 |  |
| IL-1β | 0.80 (0.60–1.24) | 2.44 (1.96–3.07) | 2.31E-07 | **1.27E-06** | ↑ |
| IL-18 | 22.17 (13.84–30.70) | 0.77 (0.51–1.26) | 2.54E-09 | **2.79E-08** | ↓ |
| IL-2Rα | 23.99 (18.10–28.20) | 43.50 (32.85–53.73) | 2.53E-06 | **1.24E-05** | ↑ |
| IL-4 | 0.94 (0.76–1.13) | 1.19 (0.94–1.38) | 0.012 | **0.023** | ↑ |
| IL-10 | 1.55 (1.14–1.78) | 1.55 (1.23–2.73) | 0.345 | 0.433 |  |
| IL-7 | 6.72 (5.44–8.27) | 5.85 (4.94–8.27) | 0.472 | 0.577 |  |
| IL-12p40 | 21.24 (18.05–27.55) | 25.09 (21.53–30.48) | 0.013 | **0.023** | ↑ |
| IL-12p70 | 0.50 (0.43–0.57) | 0.47 (0.34–0.69) | 0.963 | 0.963 |  |
| TNF-α | 14.50 (11.84–15.71) | 14.32 (12.11–17.05) | 0.68 | 0.787 |  |
| TNF-β | 307.08 (289.34–331.33) | 313.81 (277.07–325.95) | 0.942 | 0.963 |  |
| IL-9 | 313.88 (300.57–334.95) | 300.57 (271.20–341.37) | 0.128 | 0.201 |  |
| IL-13 | 0.81 (0.64–1.34) | 0.70 (0.50–0.96) | 0.344 | 0.433 |  |
| IL-15 | 0.01 (0.00–0.02) | 0.18 (0.15–0.37) | 5.01E-10 | **1.69E-08** | ↑ |
| IL-16 | 21.46 (18.14–26.37) | 26.37 (20.66–34.08) | 0.111 | 0.181 |  |
| IL-17 | 3.55 (3.29–4.20) | 3.76 (3.11–4.42) | 0.847 | 0.909 |  |
| IL-2 | 0.41 (0.37–0.47) | 0.31 (0.23–0.44) | 0.015 | **0.026** | ↓ |
| **Chemokines** |  |  |  |  |  |
| Eotaxin | 9.68 (7.20–13.10) | 5.96 (5.29–7.52) | 2.00E-04 | **6.29E-04** | ↓ |
| CTACK | 116.32 (89.68–149.21) | 71.45 (61.06–87.29) | 6.09E-05 | **2.44E-04** | ↓ |
| LIF | 5.97 (4.90–9.16) | 8.92 (7.73–15.08) | 0.001 | **0.003** | ↑ |
| MCP-1 | 5.12 (3.58–5.76) | 5.83 (5.17–8.06) | 0.008 | **0.017** | ↑ |
| MCP-3 | 0.21 (0.16–0.35) | 0.36 (0.22–0.52) | 0.001 | **0.003** | ↑ |
| MIF | 106.11 (82.18–127.38) | 95.09 (74.60–109.46) | 0.153 | 0.225 |  |
| MIG | 31.06 (22.94–37.34) | 26.13 (21.96–32.96) | 0.516 | 0.613 |  |
| MIP-1α | 0.96 (0.88–1.12) | 1.29 (1.02–1.62) | 0.001 | **0.003** | ↑ |
| MIP-1β | 94.38 (90.14–97.77) | 90.87 (85.62–97.77) | 0.263 | 0.374 |  |
| RANTES | 1564.00 (1071.50–1642.25) | 1219.00 (893.80–1419.00) | 0.037 | 0.062 |  |
| IP-10 | 156.35 (120.91–195.42) | 74.11 (53.54–97.60) | 5.41E-06 | **2.38E-05** | ↓ |
| SDF-1α | 383.76 (348.26–491.03) | 284.93 (224.55–305.70) | 1.72E-07 | **1.08E-06** | ↓ |
| IL-8 | 0.77 (0.65–1.11) | 0.94 (0.62–1.13) | 0.701 | 0.790 |  |
| GRO-α | 0.78 (0.13–3.39) | 0.02 (0.00–1.02) | 0.010 | **0.022** | ↓ |
| **Growth Factors** |  |  |  |  |  |
| Basic FGF | 9.21 (7.38–10.62) | 7.62 (6.22–9.85) | 0.139 | 0.211 |  |
| G-CSF | 1.84 (1.42–2.10) | 4.28 (3.61–4.60) | 4.32E-08 | **3.17E-07** | ↑ |
| HGF | 174.41 (152.25–228.51) | 135.87 (119.31–155.63) | 1.00E-04 | **3.39E-04** | ↓ |
| M-CSF | 20.20 (15.57–23.48) | 29.48 (23.38–36.45) | 8.00E-04 | **0.002** | ↑ |
| PDGF-BB | 81.17 (44.38–117.61) | 44.87 (22.55–68.53) | 0.011 | **0.022** | ↓ |
| SCF | 25.42 (18.83–31.43) | 85.04 (65.94–94.76) | 7.70E-10 | **1.69E-08** | ↑ |
| SCGF-β | 36602.00 (26022.75–45210.50) | 35818.00 (31452.00–43409.75) | 0.791 | 0.870 |  |
| GM-CSF | 0.27 (0.23–0.39) | 0.28 (0.26–0.42) | 0.905 | 0.948 |  |
| TRAIL | 9.30 (8.34–11.30) | 48.25 (30.69–63.24) | 2.27E-09 | **2.79E-08** | ↑ |

All data are presented as median with IQR. Bold values represent significantly different cytokines, chemokines and growth factors between maternal peripheral blood (PB) and fetal cord blood (CB). Statistical analysis was performed using the Mann–Whitney U test, and P values were adjusted for multiple comparisons using the false discovery rate (FDR) correction (Benjamini–Hochberg method).

**Supplementary Table 4. Comparative trends of immune mediators in fetal cord blood relative to maternal peripheral blood across term, S-PTB, and PROM-PTB groups.**

| **Immune mediators** | **Term** | **S-PTB** | **PROM-PTB** |
| --- | --- | --- | --- |
| **Cytokines** |  |  |  |
| IFN-α2 |  | ↓ |  |
| IL-6 | ↓ | ↓ | ↓ |
| IFN-γ |  | ↑ | ↑ |
| IL-1ra |  |  |  |
| IL-1β | ↑ | ↑ | ↑ |
| IL-18 | ↓ | ↓ | ↓ |
| IL-2Rα | ↑ | ↑ | ↑ |
| IL-4 | ↑ |  | ↑ |
| IL-10 |  |  |  |
| IL-7 |  | ↓ |  |
| IL-12p40 | ↑ | ↑ | ↑ |
| IL-12p70 |  |  |  |
| TNF-α |  |  |  |
| TNF-β |  | ↑ |  |
| IL-9 |  | ↑ |  |
| IL-13 |  |  |  |
| IL-15 | ↑ | ↑ | ↑ |
| IL-16 |  |  |  |
| IL-17 |  |  |  |
| IL-2 |  | ↓ | ↓ |
| **Chemokines** |  |  |  |
| Eotaxin |  |  | ↓ |
| CTACK |  |  | ↓ |
| LIF | ↑ |  | ↑ |
| MCP-1 |  | ↑ | ↑ |
| MCP-3 |  |  | ↑ |
| MIF |  |  |  |
| MIG |  |  |  |
| MIP-1α |  | ↑ | ↑ |
| MIP-1β |  | ↑ |  |
| RANTES |  |  |  |
| IP-10 | ↓ | ↓ | ↓ |
| SDF-1α | ↓ | ↓ | ↓ |
| IL-8 |  |  |  |
| GRO-α |  |  | ↓ |
| **Growth Factors** |  |  |  |
| Basic FGF |  |  |  |
| G-CSF | ↑ | ↑ | ↑ |
| HGF |  | ↓ | ↓ |
| M-CSF |  | ↑ | ↑ |
| PDGF-BB |  |  | ↓ |
| SCF | ↑ | ↑ | ↑ |
| SCGF-β |  | ↑ |  |
| GM-CSF |  |  |  |
| TRAIL | ↑ | ↑ | ↑ |
